# Anterior knee pain and its extrinsic risk factors among runners in under-resourced communities in Ekurhuleni, Gauteng, South Africa

**DOI:** 10.17159/2078-516X/2019/v31i1a6090

**Published:** 2019-01-01

**Authors:** S H Kunene, S Ramklass, N P Taukobong

**Affiliations:** 1 Physiotherapy Department, University of the Witwatersrand, Johannesburg, South Africa; 2 School of Clinical Medicine, University of KwaZuluNatal, Durban, South Africa; 3 Institutional Planning Department, Sefako Makgatho Health Sciences University, Pretoria, South Africa

**Keywords:** patellofemoral pain, predisposing factors, athletes, poor resourced communities

## Abstract

**Background:**

Various factors predispose athletes to anterior knee pain (AKP), making a holistic assessment with rehabilitation inevitable. Due to minimal rehabilitation services in under-resourced communities, runners are less likely to report this injury to medical professionals compared to runners in better resourced communities.

**Objective:**

The purpose of this study was to report on the prevalence of AKP among runners in under-resourced communities and to determine the extrinsic risk factors for this injury.

**Methods:**

This was a cross-sectional study of 347 runners in total. Convenience sampling was used to recruit 183 participants aged between 13 and 55 years with no previous history of knee surgeries, traumatic or degenerative knee conditions. Questionnaires were used to collect data on the prevalence of AKP and extrinsic risk factors. The SPSS (version 25) was used to analyse the data. Data were presented as frequencies and percentages and the results from chi-square and logistic regression tests were provided.

**Results:**

Forty percent (40%) of participants presented with AKP, particularly males (n=106, 58%), young runners (n=94, 51%) and those with 3–5 years of running experience (n=57, 31%). Anterior knee pain was associated with age (X^2^=6.484, *p*=0.039) and running experience (X^2^=8.39, *p*=0.04). The following extrinsic risk factors contributed to AKP significantly: training load (p=0.04, odds ratio [OR]=1.23), warm-up (p=0.04, OR=1.57)’ running shoe condition (p=0.04, OR=0.14) and running surface (p=0.05, OR=1.2).

**Conclusion:**

A substantial presence of AKP and its extrinsic risk factors were found among all participants. These outcomes suggest that extrinsic risk factors should also be considered when managing AKP among runners.

Anterior knee pain (AKP) is the most problematic symptom among many runners worldwide (15–45%), with females, adolescents and young adults being the most affected.^[1,2]^ Runners usually describe AKP as pain on the anterior aspect of the knee, beneath or around the edges of the patellar. Their pain is usually triggered or made worse by running, squatting, going up and down stairs, cycling and jumping activities. Anterior knee pain is a consequence of overuse but can also be experienced after an acute and traumatic injury, such as falling on the knee. Any activity that requires consistent compression force on the patellofemoral joint may trigger this kind of pain. The cause of AKP is usually multifactorial.^[3]^

Anterior knee pain has a negative impact on the quality of life (QOL) of many athletes. Athletes with AKP may experience various problems such as physical limitations, emotional and social difficulties which may have a detrimental effect on their well-being and ability to perform optimally.^[4]^ These experiences may also affect athletes’ participation in their rehabilitation programmes and their return to sport. Apart from the physical features, anterior knee pain also has non-physical features which may influence the recovery of patients. Theses non-physical characteristics may include psychosocial-, emotional and mental features. A previous study highlighted the negative impact of AKP on the QOL of runners from under-resourced communities, recommending the need to address the non-physical features when formulating strategies to improve QOL among the running population with AKP.^[5]^

Runners can be predisposed to AKP through intrinsic and external factors. Intrinsic factors may include a weak vastus medialis oblique, tight gastrocnemius-soleus muscle complex, dysfunction of the hip muscles, foot pronation, generalised joint laxity, limb length discrepancy, patellar malalignment and patellar hypermobility.^[6]^ In particular, tight hamstrings and weakness of the iliotibial band and quadriceps, weak hip control muscles and patellar tilt abnormalities were found to be significant when associated with AKP in an under-resourced community in South Africa.^[7]^ It is therefore critical that the intrinsic risk factors be considered in the management of AKP in order to improve management outcomes.^[7]^

Establishing extrinsic risk factors is another important component to be considered when dealing with the management of AKP. Extrinsic factors which are external to the body may include: the action of running, the level of participation (including training and competition), other types of physical activities and the way they are performed, running surfaces, environmental conditions, and the effect of the equipment encountered during physical activities, e.g. air resistance, gravity and ground reaction forces, and shoes.^[2]^ No previous studies have reported on the association between extrinsic risk factors and AKP in runners from under-resourced South African communities.

South African healthcare is challenged due to the lack of health resources and scarce professional skills.^[8]^ Most healthcare facilities in rural or peri-urban communities do not have rehabilitation healthcare professionals, which results in patients not receiving adequate and holistic rehabilitation services. According to these authors’ experience within physiotherapy clinical settings, injuries associated with AKP are experienced by many runners from these under-resourced communities, which often results in the end of their running careers due to lack of rehabilitation services. This in turn leads to poor QOL for these runners.

This study’s objective was therefore to determine the prevalence of AKP and its extrinsic risk factors among runners in under-resourced communities in Ekurhuleni, Gauteng province, South Africa.

## Methods

The population for this cross-sectional study included 183 out of 347 long-distance recreational runners from six developing clubs in under-resourced, peri-urban communities in Ekurhuleni, Gauteng province, South Africa. The same methodology was used as in the authors’ previous study.^[7]^ These runners were aged between 13 and 55 years with no history of knee surgery, traumatic or degenerative knee conditions. As in a previous study by these authors a convenience sampling method was used.^[7]^ Runners were recruited during their training sessions from six different clubs. A Raosoft statistical tool was used to calculate the study’s sample size of 183 runners, taking into consideration a 95% confidence level, 5% margin of error and 50% response distribution (http://www.raosoft.com/samplesize.html). Participants were recruited during their training sessions at various training grounds in their areas.

Self-administered questionnaires were used to collect demographic profiles and the presence of AKP and extrinsic risk factors, as described in the previous study.^[7]^ Demographic data included gender, age, running experience, and height and weight to calculate their body mass index (BMI). A standardised AKP questionnaire by Kujala and colleagues was utilised to determine the prevalence of AKP. It consisted of 13 short questions about the participant’s knee symptoms and functional limitations associated with AKP.^[9]^ The tool has a maximum score of 100 which was used to rate all participants’ AKP symptoms. A cut-off score of 83 identified participants with AKP as recommended by Kujala and colleagues. This standardised AKP questionnaire has good test-retest reliability and high validity (ICC = 0.92).^[9,10]^

The extrinsic risk factor questionnaire was developed by using the current literature available. It was reviewed by five independent experts for content validity. The reviewers included local senior clinicians and researchers in the field of musculoskeletal injuries. Following the review process, a pilot study was conducted among the same population (10% of the sample size) to check its validity. No changes or revisions were made to the questionnaire after the pilot study.

This study obtained ethical clearance from the biomedical research ethics committee of the University of KwaZulu-Natal (BFC377/15). Before the data were collected, all participants were given leaflets providing details about the study and they were requested to complete consent forms once they agreed to participate in the study. Consent was obtained from parents/guardians of participants younger than 18 years. During the data collection, the first author hand-delivered the questionnaires to the participants during their training sessions and collected them immediately after completion.

Data were captured using Microsoft Excel first and later imported into SPSS for analysis. Descriptive statistics included the calculation of frequencies and percentages for single variables. Inferential statistics included the calculation of the chi-square to determine the association between variables. Logistic regression analysis was done to describe data and explain the relationship between AKP and various independent variables (risk factors). The level of significance was set at p ≤ 0.05.

## Results

All 183 participants completed the questionnaires. Anterior knee pain was present among 40% of the participants. Participants who scored ≤83 points were considered to have AKP, according to the Kujala scoring guide ^[9]^. This study was dominated by males (n = 106, 58%), followed by young people (n = 94, 51%) and thereafter participants with 3–5 years of running experience (n = 57, 31%) (Table 1). Most participants presented with a normal BMI (n = 110, 60%).

Anterior knee pain was found to be strongly associated with age (**χ**^2^ = 6.48, p = 0.04) followed by the group of young people (n = 33, 18%) and, lastly, running experience (**χ**^2^ = 8.39, p = 0.04). Participants with 1–3-years of running experience was the most affected group (n = 22, 12%) (Table 2).

The results in Table 3 pertain to the participants’ level of training, competition and skills. Most participants were involved in endurance training (n = 167, 91%) which included mostly running. Fewer participants included strength (n = 73, 40%) and flexibility (n = 68, 37%) training in their training sessions. Most participants (n = 125, 68%) trained twice or more per week completing 16 – 21 km (n =54, 30%) for more than 60 minutes per training session (n = 82, 45%). Fifty-nine (32%) participants participated in competitions a few times a year, followed by those who participated in competitions between 1–2 times per month (n = 51, 28%). Distances covered by most participants during competition were 33–42 km (n = 42, 23%), 22–32 km (n = 37, 20%), 16–21 km (n = 35, 19%) and 10–15 km (n = 32, 18%) respectively. Only 67 (37%) participants always included a warm-up during and following their training, while most participants regularly included a warm-up (n = 111, 61%) during their training and competition.

Table 4 includes details of the running equipment used for the participants. Many participants used running shoes during training and competition (n = 172, 94%) and a few ran barefoot (n = 11, 6%). Stability shoes for neutral pronators (n = 102, 56%) were mostly used, followed by cushioning shoes for supinators (n = 20, 11%). Approximately half of the participants reported that their shoes were in good condition (n = 90, 61%). Fifty-nine (32%) participants reported that their shoes were slightly damaged and worn-out (fair condition). However, thirty-four participants (19%) reported that their shoes were completely damaged or worn-out. Only 33 (18%) participants used orthotic inserts and some used arch inserts (n = 11, 6%). About 33 (18%) reported that they used braces and of these, 17 (9%) used knee braces. Soft braces were used among the participants who used braces (n = 24, 13%).

Participants mostly trained (n = 123, 67%) and competed (n = 149, 81%) on concreate or tar surfaces (Table 5). Most participants sometimes ran downhill (n = 132, 72%) and uphill (n = 145, 79%) that were mostly dry (n = 104, 57%) while others ran on mixed surfaces (sometimes dry and wet) (n = 73, 40%). Nearly all of the participants ran in variegated weather conditions (hot, warm or cool) (n = 98, 54%).

The binary logistic regression of extrinsic risk factors to AKP among the participants is represented in Tables 6a and 6b. The model used showed 35% (Nagelkerke *R^2^*) of the variance in AKP and correctly classified 71% of cases. The following factors contributed significantly to AKP: training load (p = 0.04, odds ratio = 1.23); warm-up (p = 0.04, odds ratio = 1.57); running shoe condition (p = 0.04, odds ratio = 0.14); downhill run (p = 0.05, odds ratio = 1.20). Other factors did not show any significant contribution to AKP among participants.

## Discussion

This study described the a higher prevalence of AKP among this population of runners compared to the global prevalence which is between 15–45% according to Cook el al.^[1]^ Young and inexperienced runners were more affected compared to the rest of the population. These results were found to be congruent with other studies.^[1,11]^ The causes of AKP have been found to be multifactorial. This section will further discuss the extrinsic risk factors presented in the Results section above. Various factors were described in this study some of which contributed to AKP significantly.

Training load is one of the factors reported in the literature as a contributor to many sports-related injuries including AKP.^[12]^ Both underloading and overloading can put a runner at risk of injuries and low performance. In this study, participants who trained two or more times a week were less likely to develop AKP compared to those who never trained or trained inadequately. This undertraining or lack of training contributed to many of the participants experiencing AKP during running races. According to a study by Nielson et al., runners who trained for competition regularly were found to be less likely to have running-related injuries than those who did not train.^[12]^ Many injuries are as a result of muscle fatigue which may lead to muscle imbalances. Therefore, adequate training lowers muscle fatigue thresholds and allow a runner to perform better. Training load and fatigue should always be monitored and modified during training and competition so that injury risks can be lowered.

Overtraining syndrome is a common problem among athletes in general. It consists of “prolonged fatigue and underperformance, following a period of heavy training or competition, lasting at least two weeks.”^[13]^ As much as overtraining is a good technique to improve performance among athletes, it can lead to bodily harm and underperformance, putting strain on the body and supressing the immune system. This may lead to risks of injuries and illnesses. Therefore, it is important for runners to avoid overtraining when preparing for their running races. A runner’s fitness levels, body composition, level of running, injury history and age should be considered when determining training loads. New runners tend to do too much too soon. They should rather consider to increase their training load gradually in order to avoid overuse injuries.

This study reported that many participants occasionally included warm-up and cool-down sessions during training and competition. However, warm-up and cool-down factors did not show any significant contribution to AKP. Warm-up is a low-level activity which prepares the body for vigorous activity. In order to obtain optimal benefits and reduce injury risks, runners should include specific warm-up exercises in their training programmes.^[14]^ A well-structured and targeted warm-up programme will also reduce the risks of AKP in runners. A warm-up programme may include 10–15 minutes of low-level physical activities that mimic the main activity (e.g. walking, slow-paced running etc.) and lower limb dynamic stretching and strengthening exercises. A cool-down is also an important part of training and competition, which allows the body to make a smooth transition from the main activity and a state of rest. A cool-down may include the same kind of low-level physical activities as in the warm-up and lower limb static stretching.

Running shoes is another critical factor that runners and those training them should consider in order to prevent injuries. This study reported that many participants used running shoes, but more than half participants reported that their shoes were not in good condition. Runners with poor running shoes were found to be more likely to get AKP. A few runners used shoe inserts (mostly foot arch inserts). It is therefore necessary for runners to get assessed for the appropriate footwear for their gait in order to reduce the risks of AKP. Footwear should be appropriate for the runner’s foot type. Saxena and Haddad reported that approximately 76% of their study’s participants benefited greatly from orthotics during their AKP rehabilitation.^[16]^ Orthotics helps to limit the maximum amount of unwanted foot pronation, reduce the speed of internal tibial rotation by reducing the amount of sudden stresses applied to the under surface of the patella. It would be unnecessary for the vastus medialis oblique (VMO) to work so hard in maintaining proper tracking and positioning of the patella.^[15]^ Brukner and Khan supports the use of soft foot orthoses or rigid orthoses especially if the subtalar joint and tibial rotations are a problem and intervention is required.^[2]^

Use of braces is another factor that is considered to be a contributor to the prevention of AKP. According to this study very few runners used braces and those who did, used soft knee braces. As much as knee braces are used by athletes to prevent and treat AKP, there is lack of evidence on the benefit of their use. For example, there is little evidence in the literature supporting the use of knee orthoses for pain reduction in athletes presenting with AKP.^[16]^ Further research is necessary on the use of knee orthoses in treating AKP.

Running surface is another risk factor for injuries. Most runners in this study ran on hard concrete/tar surfaces, some of which were downhill. Running on a hard surface and going downhill were risks for AKP. Runners should vary the surfaces they run on to prevent AKP injuries.

Varying exercises is another way to limit the risks of AKP. For example, they should consider alternating running with biking, swimming etc. thereby exerting less stress on the knees.

## Conclusion

This study reported a substantial number of AKP injuries, particularly among males, young runners and those with 3–5 years of running experience. Various extrinsic risk factors were identified and the following were found to have contributed significantly to AKP among this study’s population: training load, shoe condition, running surface especially running downhill on a hard surface. The outcome of this study suggests a need for the development of prevention, treatment and rehabilitation programmes to address the problems described among runners in poorly resourced communities. To overcome the problem of limited healthcare professional services in many communities in South Africa, community-based rehabilitation programmes are highly recommended for runners. Therefore a further study is needed to develop AKP community-based rehabilitation programmes and a framework for its implementation to assist in the problems facing runners in under-resourced communities.

## Figures and Tables

**Table 1:** Demographics profile of runners (n = 183)

Demographics	Categories	N	%
**Gender**	Male	106	58
	Female	77	42
**Age (years)**	13–17	51	28
	18–35	94	51
	36–55	38	21
**Running experience**	<1 year	20	11
	1–3 years	49	27
	3–5 years	57	31
	6–10 years	37	20
	>10 years	20	11
**BMI**	<18.5	28	15
	18.5–24.9	110	60
	25–29.9	42	23
	>30	3	2

Kunene et al.^[7]^

**Table 2:** Anterior knee pain (AKP) and demographic profile (n = 183)

Demographics	Categories	AKP n (%)	Chi-square and p-values
**Gender**	Male	40 (22%)	**χ2 = 0.49**
	Female	33 (18%)	p = 0.49
**Age (years)**	13–17	18 (10%)	
	18–35	33 (18%)	**χ2 = 6.48**
	36–55	22 (12%)	p = 0.04
**Running experience**	<1 year	7 (4.8%)	
	1–3 years	22 (12%)	
	3–5 years	19 (10.4%)	**χ2 = 8.39**
	6–10 years	12 (6.6%)	p = 0.04
	>10 years	13 (7.1%)	
**BMI**	<18.5	12 (6.6%)	
	18.5–24.9	44 (24%)	**χ**2 = 5.38
	25–29.9	14 (7.7%)	p = 0.15
	>30	3 (1.6%)	

Kunene et al.^[7]^

**Table 3:** Level of training, competition and skills (n = 183)

Factors	N (%)
**Training methods**	
Strength	73(40)
Endurance	167(91)
Stretching	68(37)
Aerobic	47(26)
Other	24(13)
**Training frequency**	
Never	9(5)
1–3 times/month	33(18)
Once a week	16(9)
≥2 times/week	125(68)
**Training duration**	
5–10 min	7(4)
11–20 min	5(3)
21–40 min	43(24)
41–60 min	46(25)
>60 min	82(45)
**Running distance covered during training**	
≤5 km	20(11)
10–15 km	50(27)
16–21 km	54(30)
22–32 km	32(18)
33–42 km	25(14)
≥42 km	2(1)
**Race frequency**	
Few times a year	59(32)
Once a month	38(21)
2–3 times/month	51(28)
Once a week	20(11)
≥2 a week	15(8)
**Running distance covered during competition**	
≤5 km	11(6)
10–15 km	32(18)
16–21 km	35(19)
22–32 km	37(20)
33–42 km	42(23)
≥42 km	26(14)
**Warm-up during training/competition**	
Always	65(36)
Sometimes	111(61)
Never	7(4)
**Cool-down during training/competition**	
Always	67(37)
Sometimes	97(53)
Never	19(10)

**Table 4:** Running equipment (n = 183)

Factors	N (%)
**Use of running shoes**	
Yes	172(94)
No	11(6)
**Types of shoes used**	
Stability	102(56)
Motor control	7(4)
Cushioning	20(11)
Other	43(24)
**Shoe condition**	
Good	90(49)
Fair	59(32)
Poor	34(19)
**Use of orthotic inserts**	
Yes	33(18)
No	150(82)
**Type of orthotic inserts used**	
Heel	4(2)
Arch	11(6)
Ball of foot	7(4)
Not sure	11(6)
**Use of braces**	
Yes	33(18)
No	150(82)
**Body part that use braces**	
Knee	17(9)
Ankle	2(1)
Both	14(8)
**Type of braces**	
Soft	23(13)
Hard	10(6)

**Table 5:** Running surface and environment (n = 183)

Factors	N (%)
**Training surface**	
Grass	17(9)
Earth	13(7)
Concrete/tar	123(67)
Mixed	30(16)
**Competition surface**	
Grass	6(3)
Earth	5(3)
Concrete/tar	149(81)
Mixed	23(13)
**Downhill run**	
Mostly	39(21)
Sometimes	132(72)
Never	12(7)
**Uphill run**	
Mostly	38(21)
Sometimes	145(79)
**Surface condition (training/competition)**	
Dry	104(57)
Wet	6(3)
Mixed	73(40)
**Weather conditions (training/competition)**	
Hot	7(4)
Warm	41(22)
Cool	37(20)
Mixed	98(54)

**Table 6a:** Logistic regression of extrinsic risk factors (n = 183)

Factors	Categories	p-values	Odds ratio	95% C.I.
**Running experience**	<1 year	1		
	1–3 years	0.65	0.72	0.17 – 1.02
	3–5 years	0.53	1.59	0.38 – 1.72
	6–10 years	0.74	0.78	0.18 – 1.93
	>10 years	0.11	0.26	0.05 – 1.37
**Training load**	Never	1		
	1–3 times/month	0.10	1.22	0.47 – 1.59
	Once a week	0.58	1.37	0.04 – 1.92
	≥2 times/week	0.04*	1.23	0.66 – 1.29
**Competition frequency**	Few times a year	1		
	Once a month	0.64	1.46	0.30 – 1.04
	2–3 times/month	0.45	1.66	0.45 – 1.12
	Once a week	0.66	0.58	0.05 – 1.68
	≥2 a week	0.96	0.93	0.05 – 1.12
**Training distance**	≤5 km	1		
	10–15 km	0.51	0.37	0.02 – 1.42
	16–21 km	0.85	1.30	0.080 – 1.94
	22–32 km	0.71	0.57	0.030 – 1.90
	33–42 km	0.94	0.87	0.03 – 1.14
	≥42 km	0.70	0.66	0.02 – 1.81
**Competition distance**	≤5 km	1		
	10–15 km	0.09	0.06	0.00 – 1.54
	16–21 km	0.19	0.02	0.00 – 1.03
	22–32 km	0.27	0.14	0.00 – 1.79
	33–42 km	0.22	0.10	0.00 – 1.77
	≥42 km	0.21	0.11	0.00 – 1.36
**Warm-up**	Always	1		
	Sometimes	0.04*	1.57	0.39 – 1.97
	Never	0.53	1.15	0.25 – 1.71
**Cool-down**	Always	1		
	Sometimes	0.36	0.48	0.10 – 1.80
	Never	0.44	1.70	0.21 – 1.99
**Running with shoes**	Yes			
	No	0.90	0.74	0.01 – 1.07

* indicates significant p value < 0.05

**Table 6b:** Logistic regression of extrinsic risk factors (n = 183)

Factors	Categories	p-values	Odds ratio	95% C.I.
**Shoe condition**	Good	1		
	Fair	0.56	0.30	0.01 – 1.73
	Poor	0.04*	0.14	0.00 – 1.99
**Use of orthotic insert**	Yes	1		
	No	0.10	0.15	0.02 – 1.42
**Use of braces**	Yes	1		
	No	0.11	0.66	0.72 – 1.10
**Training surface**	Grass	1		
	Earth	0.12	0.05	0.00 – 1.06
	Concrete/tar	0.77	0.72	0.08 – 1.57
	Mixed	0.10	0.10	0.01 – 1.54
**Competition surface**	Grass	1		
	Earth	0.29	1.75	0.10 – 1.77
	Concrete/tar	0.66	2.43	0.05 – 1.12
	Mixed	0.35	8.68	0.09 – 1.11
**Running downhill**	Mostly	1		
	Sometimes	0.05*	1.20	0.97 – 1.96
	Never	0.15	0.30	0.94 – 1.83
**Running uphill**	Mostly	1		
	Sometimes	0.54	0.32	0.01 – 1.22
	Never	0.45	0.46	0.00 – 1.48
**Running surface**	Dry			
	Wet	0.79	0.40	0.02 – 1.35
	Mixed	0.58	1.52	0.35 – 1.58
**Weather conditions**	Hot	1		
	Warm	0.40	1.25	0.01 – 1.34
	Cool	0.45	1.82	0.12 – 1.71
	Mixed	0.27	0.17	0.01 – 1.87

* indicates significant p value < 0.05
